# Epidemiology of urinary *Escherichia coli* from a population-based surveillance in three U.S. counties, August 2023

**DOI:** 10.1017/ash.2026.10809

**Published:** 2026-10-06

**Authors:** Joshua M. Brandenburg, Latasha Curtis, Ruben E. Raymond, Uzma Ansari, Davina E. Campbell, Amy S. Gargis, Alyssa G. Kent, Susannah L. McKay, Erin C. Parker, Rebecca Longson, Gillian Smith, Jesse T. Jacob, Paulina A. Rebolledo, Ghinwa Dumyati, Hsioa Che Looi, Julia Tellerman, Alice Y. Guh, Heather N. Grome

**Affiliations:** 1 Division of Healthcare Quality Promotion, Centers for Disease Control and Prevention, Atlanta, USA; 2 California Emerging Infections Program, Oakland, USA; 3 Georgia Emerging Infections Program, Atlanta, USA; 4 Emory University School of Medicine, Atlanta, USA; 5 New York Emerging Infections Program and University of Rochester Medical Center, Rochester, USA

## Abstract

Population-based surveillance of urinary *Escherichia coli* was conducted in three U.S. counties. Findings included high incidence among females and older adults, resistance to recommended urinary tract infection treatments, and O serotypes common among invasive disease. The study emphasizes the value of understanding the epidemiology and resistance profiles to guide treatment.

## Introduction

*Escherichia coli* is a leading global cause of urinary tract infections (UTIs) including complications such as pyelonephritis, bacteremia, and community-onset sepsis.^1^ Additionally, increasing rates of antibiotic resistance in urinary *E. coli* (uEc) substantially increase the societal burden of these common infections.^2^

Recent U.S. surveillance rarely characterizes the full epidemiology of community-associated uEc, particularly across all antimicrobial susceptibility phenotypes.^3^ This pilot expands understanding of uEc burden by providing recent, population-based data and broad O-serotype characterization across all phenotypes through one month of active surveillance conducted by the Centers for Disease Control and Prevention’s (CDC’s) Emerging Infections Program (EIP).

## Methods

Population- and laboratory-based surveillance for uEc was conducted during one month (August 2023), due to the resource-intensive nature of the surveillance, in three U.S. counties (Alameda County, California; Fulton County, Georgia; and Monroe County, New York) with a total estimated population of 3.4 million people.

Cases were identified through automated testing instruments (ATIs) or laboratory information systems of all urine cultures from the clinical laboratories serving residents of the three surveillance counties. We defined a case as the first *E. coli* of any phenotype isolated from urine in a resident of the surveillance counties over a 30-day period. Data collectors at each site recorded demographics on all case-patients and attempted to review medical records of a random sample of 400 cases to collect data on underlying conditions, healthcare exposures, and presence of UTI, bacteremia, and septic shock using a standardized case report form (CRF) (see supplementary materials). We assigned cases epidemiologic classifications: community-associated, healthcare-associated community-onset, or hospital-onset (see supplementary materials).

Minimum inhibitory concentrations from antimicrobial susceptibility testing (AST) results from clinical lab ATIs or from the medical record were collected on the CRF. Carbapenem resistance was defined as resistance to ≥1 carbapenem and extended-spectrum β-lactamase (ESBL)-production as resistance to ≥1 extended-spectrum cephalosporin and without carbapenem resistance. For cases with completed CRFs, an isolate was submitted to CDC for whole genome sequencing (WGS) analysis and *in silico* serotyping at the CDC laboratory (see supplementary materials).

We calculated one-month incidence rates per 100,000 population using aggregate 2023 U.S. Census county population estimates. Incidence rates by demographic subgroups were calculated using stratum-specific denominators. We conducted data analyses using R v4.4.0 (https://cran.r-project.org/).

This activity was reviewed by CDC, deemed not research, and was conducted consistent with applicable federal law and CDC policy (See for example 45 C.F.R. part 46.102(l)(2), 21 C.F.R. part 56; 42 U.S.C. §241(d); 5 U.S.C. §552a; 44 U.S.C. §3501 et seq.). This activity was also reviewed by participating EIP sites, and either was deemed non-research or received IRB approval with a waiver of informed consent.

## Results

We identified 5,742 cases in August 2023 with a crude one-month incidence rate of 166.4 per 100,000 population (Table 1). Incidence was higher among females than males (280.0 vs 44.6 per 100,000), higher among infants (<1 yr) than children aged 1–17 years (79.9 vs 39.1 per 100,000), and increased with age among adults, ranging from 117.7 per 100,000 in adults aged 18–39 years to 802.5 per 100,000 in those aged ≥80 years old (Table 1).

**Table 1.** Demographic characteristics and associated incidence rates and clinical characteristics of urinary *Escherichia coli* cases, 3 U.S. Counties, August 2023 Table 1 long description.

	No. Cases	% Cases	One-month incidence rate per 100,000	2023 Census population estimates
**Total cases**	5,742	100	166.4	3,449,775
**Cases with completed case report forms**	1,199	20.9	—	—
**Demographic characteristics among total cases (n = 5,742)**				
**Patient sex**				
Female	4,922	85.7	280.0	1,758,111
Male	755	13.1	44.6	1,691,664
Missing value	65	1.1	—	—
**Race**				
White	2,737	47.7	151.4	1,808,280
Black	1,087	18.9	139.1	781,229
Other^a^	523	9.1	60.8	860,266
Unknown	1,395	24.3	—	—
**Ethnicity**				
Non-Hispanic or Latino	4,265	74.3	146.7	2,907,857
Hispanic or Latino	739	12.9	136.4	541,918
Unknown	738	12.9	—	—
**Age** ^b^				
<1 year	29	0.5	79.9	36,301
1–17 yr	255	4.4	39.1	652,897
18–39 yr	1,298	22.6	117.7	1,102,778
40–59 yr	1,242	21.6	137.3	904,477
60–79 years	1,921	33.5	305.3	629,205
80 years +	996	17.3	802.5	124,117
**Clinical characteristics among cases with completed case report forms (n = 1,199)**				
**Epidemiologic classification** ^c^				
Community-associated	869	72.5		
Healthcare-associated, community-onset	325	27.1		
Hospital-onset	3	0.3		
Unknown	2	0.2		
**Charlson Comorbidity Index, median (range)**	0 (0–10)			
**Underlying conditions** ^d^				
None	365	30.4		
Recurrent urinary tract infection^e^	269	22.4		
Neurologic condition^f^	241	20.1		
Diabetes	239	19.9		
Obesity	227	18.9		
Urinary tract problems/abnormalities	209	17.4		
Chronic pulmonary disease	202	16.8		
Cardiovascular disease^g^	176	14.7		
Chronic kidney disease	142	11.8		
Gastrointestinal disease^h^	93	7.8		
Malignancy^i^	87	7.3		
Dementia	67	5.6		
Pregnant	60	5.0		
Connective tissue disease	44	3.7		
Skin Condition^j^	27	2.3		
Immunocompromised condition	25	2.1		
Liver disease	24	2.0		
Plegia/paralysis	10	0.8		
**Associated infection types** ^k^				
Urinary tract infection^l^	1,116	93.1		
Pyelonephritis^m^	70	5.8		
Bacteremia	43	3.6		
Septic Shock	9	0.8		
**Signs and symptoms associated with urine cultures**				
Dysuria	510	42.5		
Frequency	421	35.1		
Urgency	273	22.8		
Costovertebral angle pain or tenderness	158	13.2		
Suprapubic tenderness	157	13.1		
Fever [temperature ≥ 100.4 °F (38°C)]	96	8.0		
None of the above or unknown^n^	395	32.9		
**Colony counts associated with urine cultures**				
≥100,000 CFU/mL	867	72.3		
<100,000 CFU/mL^o^	318	26.5		
Unknown	14	1.2		

a Other: Includes Asian, American Indian or Alaskan Native, Native Hawaiian or Other Pacific Islander, or ≥2 races reported.

b Patient age missing for one case.

c Epidemiologic Classification defined as Hospital-onset: Urine obtained ≥ 3 days after hospital admission; Healthcare-associated, community-onset: Urine obtained in a non-hospital setting or<3 days after hospital admission with selected healthcare exposures (hospitalization, surgery, residence in a long-term care facility or long-term acute care hospital) in the prior year, chronic dialysis, or with an indwelling device in the 2 days prior to culture; Community-associated: Urine obtained in a case-patient without any of the healthcare risk factors described.

d Cases may have>1 underlying condition reported.

e Recurrent UTI as documented in the medical record.

f Neurologic Condition includes cerebral palsy, chronic cognitive deficit, epilepsy/seizure/seizure disorder, multiple sclerosis, neuropathy, Parkinson’s disease, and others.

g Cardiovascular disease includes cerebrovascular accident, transient ischemic attack, congenital heart disease, congestive heart failure, myocardial infarction, and peripheral vascular disease.

h Gastrointestinal disease includes diverticular disease, inflammatory bowel disease, peptic ulcer disease, and short gut syndrome.

i Malignancy includes hematologic and solid organ (metastatic and non-metastatic).

j Skin condition includes burn, decubitus or pressure ulcers, surgical wound, and other chronic ulcer or chronic wound.

k Cases may have>1 associated infection type reported.

l Urinary tract infection defined as documented in the medical record.

m Pyelonephritis as documented in the medical record, and presented as a subset of the UTI count.

n Includes case-patients with no or unknown (22/1197; 1.8%) signs or symptoms or those with other signs or symptoms not listed in the table, which would not have been captured by the standardized case report form. Signs and symptoms reported are limited to those captured on the case report form.

o Colony counts reported as a range up to 100,000 CFU/ml (e.g., “10 to 100,000” was included).

Of 1,199 cases with a completed CRF, 869 (72.5%) were community-associated. Of these, 1,116 (93.1%) had UTI documented (Table 1), including 42 (3.8%) with bacteremia, and pyelonephritis was documented in 70 (5.8%) cases, including 11 (15.7%) with bacteremia. Quantitative urine culture results were available for 1,103 (98.8%) of 1,116 cases with UTI. Most (821/1,103 [74.4%]) had cultures with ≥10^5^ colony-forming-units per milliliter (CFU/mL). Among the 282 cultures < 10^5^ CFU/mL, 223 (79.1%) of 282 cases had UTI signs or symptoms (Table 1). A urinary catheter was present within two days before specimen collection for 77 (6.4%) cases.

Among cases with AST results (**eTable 1**), 1.5% (13/839) were resistant to nitrofurantoin, 63.1% (424/672) were resistant to trimethoprim-sulfamethoxazole, and 20.0% (192/961) were resistant to a fluoroquinolone (ciprofloxacin or levofloxacin). None showed resistance to all first-line recommended oral antibiotics for UTI.^4,5^ Separately, 12.3% (147/1,199) were ESBL-producing; none were carbapenem resistant.

A total of 939 isolates were submitted to CDC for WGS and *in silico* O serotyping; 100 unique serotypes were identified, most frequently O25B (115; 12.2%), O6 (98; 10.4%), and O2 (84; 8.9%) (Table 2). These three serotypes accounted for approximately 30% of the distribution of O serotypes across all reported age and sex strata. Serotype O25B was more prevalent in males than females (20.1% [28/139] vs 10.8% [86/795]), despite the increased frequency among females, and in case-patients aged ≥60 years compared with <60 years (16.1% [68/422] vs 9.1% [47/517]).

**Table 2.** Predicted O serotypes by *in silico* serotyping from whole genome sequencing of urinary *Escherichia coli* isolates tested at the U.S. Centers for Disease Control and Prevention, 3 U.S. Counties, August 2023 (n = 939) Table 2 long description.

O serotype	Total (n = 939)	%	Age < 60 yr (n = 517)	%	Age ≥ 60 yr (n = 422)	%	Male (n = 139)	%	Female (n = 795)	%
O25B	115	12.2	47	9.1	68	16.1	28	20.1	86	10.8
O6	98	10.4	54	10.4	44	10.4	13	9.4	85	10.7
O2	84	8.9	50	9.7	34	8.1	18	12.9	66	8.3
O75	69	7.3	41	7.9	28	6.6	10	7.2	57	7.2
O1A	66	7.0	38	7.4	28	6.6	7	5.0	59	7.4
O16	48	5.1	31	6.0	17	4.0	6	4.3	42	5.3
O15	28	3.0	17	3.3	11	2.6	5	3.6	23	2.9
O4	27	2.9	12	2.3	15	3.6	4	2.9	23	2.9
O8	27	2.9	13	2.5	14	3.3	3	2.2	24	3.0
O22	22	2.3	14	2.7	8	1.9	2	1.4	20	2.5
O17/O44/O77^a^	19	2.0	14	2.7	5	1.2	2	1.4	17	2.1
O46	18	1.9	8	1.5	10	2.4	1	0.7	17	2.1
O13	16	1.7	3	0.6	13	3.1	2	1.4	14	1.8
O18	16	1.7	6	1.2	10	2.4	4	2.9	12	1.5
O86	16	1.7	11	2.1	5	1.2	2	1.4	14	1.8
O153	15	1.6	13	2.5	2	0.5	1	0.7	14	1.8
O117	14	1.5	9	1.7	5	1.2	1	0.7	13	1.6
O18ac	14	1.5	8	1.5	6	1.4	2	1.4	12	1.5
O99	11	1.2	10	1.9	1	0.2	0	0.0	11	1.4
O25A	10	1.1	4	0.8	6	1.4	1	0.7	9	1.1
Other^b^	163	17.4	96	18.6	67	15.9	19	13.7	143	18.0
Unknown/not detected	43	4.6	18	3.5	25	5.9	8	5.8	34	4.3

a Closely related O serotypes are reported as a group.

b Other category includes counts of 9 or less for the following O serotypes: O7, O9, O9/O101, O9a, O10, O11, O12, O13/O129/O135, O17, O17/O44, O17/O77, O19, O21, O23, O26, O29, O32, O33, O34, O39, O45, O49, O54, O55, O61, O62, O65, O66, O78, O80, O81, O82, O83, O85, O87, O88, O89, O91, O93, O96, O98, O101, O102, O104, O105, O107, O108, O109, O112, O115, O120, O123, O124, O125ac, O128, O128ac, O131, O132, O136, O139, O145, O147, O148, O150, O152, O153/O178, O154, O157, O159, O160, O161, O163, O169, O170, O173, O175, O181, O184, O185, O188.

## Discussion

Through active population- and laboratory-based surveillance, we measured a one-month incidence rate of uEc as 166 per 100,000 population among residents of three surveillance sites, consistent with the high incidence reported in other studies based on urine culture data^6^ and nearly 27 times the rate of invasive *E. coli* infections when extrapolated to a three-month incidence rate.^7^ Incidence was six-fold higher among females compared to males, seven-fold higher among those ≥80 years old compared to 18–39 years old, and among children, incidence was higher among infants <1 year. These findings align with previously identified populations shown to have elevated uEC rates.^1,6,8^

Although the CRF did not include all possible UTI signs or symptoms, nearly one-third of cases with a CRF had none reported. This finding may indicate that urine cultures are sometimes collected without clear clinical indication.^9^ Positive urine cultures may prompt treatment without clinical evidence of UTI, leading to unnecessary antibiotics for asymptomatic bacteriuria and increased antimicrobial resistance.^9^ In addition, among those tested, nearly two-thirds of cases were trimethoprim-sulfamethoxazole resistant and one-fifth were fluoroquinolone resistant, which may limit effectiveness of these first-line recommended antibiotics to treat UTIs.^4,5^ Nearly one in eight cases were caused by ESBL-producing *E. coli*. These findings highlight the importance of appropriate urine culturing and AST of cultures to inform treatment.

Isolate testing revealed a wide diversity of O serotypes, with nearly one-third of the distribution attributable to O25B, O2, and O6. Notably, O25B, which was more common among males and case-patients 60 years and older, is often associated with the global pandemic clone sequence type 131.^10^ Furthermore, the most prevalent O serotypes in our study were similar to those reported for invasive *E. coli* infections, which are often secondary to urogenital infection.^7^ Prevention efforts aimed at reducing UTIs caused by *E. coli* may reduce rates of invasive *E. coli* bacteremia and sepsis.

This study has several limitations. First, uEc burden is likely underestimated, since our case definition required a positive culture and did not capture purely clinical or non-culture-based diagnostics; data availability from commercial laboratories also varied by state. Second, CRFs and isolate submissions were available for only a subset of cases and may not accurately reflect the overall epidemiology, resistance, or strain diversity, although random sampling was performed to minimize bias. Third, signs and symptoms may be underreported because CRF response options were not exhaustive. Fourth, one month of surveillance prevented assessment of temporal variation. Finally, findings may not be nationally generalizable, although participating counties represent three distinct U.S. regions.

## Supporting information

10.1017/ash.2026.10809.sm001 Brandenburg et al. supplementary material Brandenburg et al. supplementary material

## Supplementary material

The supplementary material for this article can be found at https://doi.org/10.1017/ash.2026.10809.

## Table 1. Long description

A table with 5,742 cases of urinary E. coli in three U.S. counties in August 2023. The table has 4 columns and 66 rows. Column headers are No. Cases, % Cases, One-month incidence rate per 100,000, and 2023 Census population estimates. Row labels include Total cases, Cases with completed case report forms, Demographic characteristics among total cases, Patient sex, Race, Ethnicity, Age, Clinical characteristics among cases with completed case report forms, Epidemiologic classification, Charlson Comorbidity Index, median (range), Underlying conditions, Associated infection types, and Signs and symptoms associated with urine cultures. Row 1: Total cases, 5,742, 100, 166.4, 3,449,775. Row 2: Cases with completed case report Forms, 1,199, 20.9, --, --. Row 3: Demographic characteristics among total cases (n = 5,742), --, --, --, --. Row 4: Patient sex, --, --, --, --. Row 5: Female, 4,922, 85.7, 280.0, 1,758,111. Row 6: Male, 755, 13.1, 44.6, 1,691,664. Row 7: Missing value, 65, 1.1, --, --. Row 8: Race, --, --, --, --. Row 9: White, 2,737, 47.7, 151.4, 1,808,280. Row 10: Black, 1,087, 18.9, 139.1, 781,229. Row 11: Other, 523, 9.1, 60.8, 860,266. Row 12: Unknown, 1,395, 24.3, --, --. Row 13: Ethnicity, --, --, --, --. Row 14: Non-Hispanic or Latino, 4,265, 74.3, 146.7, 2,907,857. Row 15: Hispanic or Latino, 739, 12.9, 136.4, 541,918. Row 16: Unknown, 738, 12.9, --, --. Row 17: Age, --, --, --, --. Row 18: <1 year, 29, 0.5, 79.9, 36,301. Row 19: 1-17 yr, 255, 4.4, 39.1, 652,897. Row 20: 18-39 yr, 1,298, 22.6, 117.7, 1,102,778. Row 21: 40-59 years, 1,242, 21.6, 137.3, 904,477. Row 22: 60-79 years, 1,921, 33.5, 309.3, 620,205. Row 23: 80 years, 996, 17.3, 802.5, 124,117. Row 24: Clinical characteristics among cases with completed case report forms (n = 1,199), --, --, --, --. Row 25: Epidemiologic classification, --, --, --, --. Row 26: Community-associated, 869, 72.5, --, --. Row 27: Healthcare-associated, community-onset, 325, 27.1, --, --. Row 28: Hospital-onset, 3, 0.3, --, --. Row 29: Unknown, 2, 0.2, --, --. Row 30: Charlson Comorbidity Index, median (range), 0 (0-10), --, --, --.

Navigate back to Table 1..

## Table 2. Long description

A table comparing the distribution of O serotypes in Escherichia coli isolates across different age and sex groups. The table has 11 columns and 22 rows. Column headers are: O serotype, Total (n = 939), %, Age < 60 yr (n = 517), %, Age ≥ 60 yr (n = 422), %, Male (n = 139), %, Female (n = 795), %. Row 1: O25B, 115, 12.2, 47, 9.1, 68, 16.1, 28, 20.1, 86, 10.8. Row 2: O6, 98, 10.4, 54, 10.4, 44, 10.4, 13, 9.4, 85, 10.7. Row 3: O2, 84, 8.9, 50, 9.7, 34, 8.1, 18, 12.9, 66, 8.3. Row 4: O75, 69, 7.3, 41, 7.9, 28, 6.6, 10, 7.2, 57, 7.2. Row 5: O1A, 66, 7.0, 38, 7.4, 28, 6.6, 7, 5.0, 59, 7.4. Row 6: O16, 48, 5.1, 31, 6.0, 17, 4.0, 6, 4.3, 42, 5.3. Row 7: O15, 28, 3.0, 17, 3.3, 11, 2.6, 5, 3.6, 23, 2.9. Row 8: O4, 27, 2.9, 12, 2.3, 15, 3.6, 4, 2.9, 23, 2.9. Row 9: O8, 27, 2.9, 13, 2.5, 14, 3.3, 3, 2.2, 24, 3.0. Row 10: O22, 22, 2.3, 14, 2.7, 8, 1.9, 2, 1.4, 20, 2.5. Row 11: O17/O44/O77, 19, 2.0, 14, 2.7, 5, 1.2, 2, 1.4, 17, 2.1. Row 12: O46, 18, 1.9, 8, 1.5, 10, 2.4, 1, 0.7, 17, 2.1. Row 13: O13, 16, 1.7, 3, 0.6, 13, 3.1, 2, 1.4, 14, 1.8. Row 14: O18, 16, 1.7, 6, 1.2, 10, 2.4, 4, 2.9, 12, 1.5. Row 15: O86, 16, 1.7, 11, 2.1, 5, 1.2, 2, 1.4, 14, 1.8. Row 16: O153, 15, 1.6, 13, 2.5, 2, 0.5, 1, 0.7, 14, 1.8. Row 17: O117, 14, 1.5, 9, 1.7, 5, 1.2, 1, 0.7, 13, 1.6. Row 18: O18ac, 14, 1.5, 8, 1.5, 6, 1.4, 2, 1.4, 12, 1.5. Row 19: O99, 11, 1.2, 10, 1.9, 1, 0.2, 0, 0.0, 11, 1.4. Row 20: O25A, 10, 1.1, 4, 0.8, 6, 1.4, 1, 0.7, 9, 1.1. Row 21: Other, 163, 17.4, 96, 18.6, 67, 15.9, 19, 13.7, 143, 18.0. Row 22: Unknown/not detected, 43, 4.6, 18, 3.5, 25, 5.9, 8, 5.8, 34, 4.3.

Navigate back to Table 2..

## References

[1] Bonten M , Johnson JR , van den Biggelaar AHJ , et al. Epidemiology of *Escherichia coli* bacteremia: A systematic literature review. Clin Infect Dis 2021;72:1211–1219. doi:10.1093/cid/ciaa210 32406495

[2] Kaye KS , Gupta V , Mulgirigama A , et al. Antimicrobial resistance trends in urine *Escherichia coli* isolates from adult and adolescent females in the United States from 2011 to 2019: rising ESBL Strains and Impact on Patient Management. Clin Infect Dis 2021;73:1992–1999. doi:10.1093/cid/ciab560 PMC8664433 34143881

[3] CDC. Multi-site gram-negative surveillance initiative (MuGSI). National Centers for Emerging and Zoonotic Infectious Diseases (NCEZID), Division of Healthcare Quality Promotion (DHQP), 2025. https://www.cdc.gov/healthcare-associated-infections/php/haic-eip/mugsi.html. Updated July 29, 2024.

[4] Gupta K , Hooton TM , Naber KG , et al. International clinical practice guidelines for the treatment of acute uncomplicated cystitis and pyelonephritis in women: A 2010 update by the infectious diseases society of America and the European Society for microbiology and infectious diseases. Clin Infect Dis 2011;52:e103–e120. doi: 10.1093/cid/ciq257 21292654

[5] Trautner BW , Cortes-Penfield NW , Gupta K , et al. Clinical practice guideline by infectious diseases society of America (IDSA): 2025 Guideline on management and treatment of complicated urinary tract infections: Duration of antibiotics for complicated UTI. Clin Infect Dis 2026;82:i79–i88. doi: 10.1093/cid/ciaf462 41419448

[6] Bruxvoort KJ , Bider-Canfield Z , Casey JA , et al. Outpatient urinary tract infections in an era of virtual healthcare: Trends from 2008 to 2017. Clin Infect Dis 2020;71:100–108. doi:10.1093/cid/ciz764 PMC7456352 31437269

[7] Grome HN , Brandenburg JM , Kent AG , et al. Extraintestinal invasive *Escherichia coli* infections in the US. JAMA Netw Open 2026;9:e2557201. doi:10.1001/jamanetworkopen.2025.57201 PMC12865657 41627814

[8] Shaikh N , Morone NE , Bost JE , Farrell MH. Prevalence of urinary tract infection in childhood: A meta-analysis. Pediatr Infect Dis J 2008;27:302–308. doi: 10.1097/INF.0b013e31815e4122 18316994

[9] Nicolle LE , Gupta K , Bradley SF , et al. Clinical practice guideline for the management of asymptomatic bacteriuria: 2019 Update by the infectious diseases society of America. Clin Infect Dis 2019;68:1611–1615. doi:10.1093/cid/ciz021 31506700

[10] Rogers BA , Sidjabat HE , Paterson DL. Escherichia coli O25b-ST131: A pandemic, multiresistant, community-associated strain. J Antimicrob Chemother 2011;66:1–14. doi:10.1093/jac/dkq415 21081548

