# Supplementary material for: Epidemiology of urinary Escherichia coli from a population-based surveillance in three U.S. counties, August 2023
Source: Antimicrob Steward Healthc Epidemiol. 2026 Oct 6;6(1):e317. doi: 10.1017/ash.2026.10809 (PMC13760402; doi:10.1017/ash.2026.10809)
Supplement: Supplementary file 1 [file S2732494X26108092sup001.docx]

**Online-Only Material**

eMethods

eTable 1

eReferences

**eMethods**

*Data Collection*

Urinary *Escherichia coli* (uEc) cases underwent medical record review using a standardized case report form (CRF) adapted from the CDC Emerging Infections Program (EIP) Multisite Gram Negative Surveillance Initiative (MuGSI) website: <https://www.cdc.gov/healthcare-associated-infections/php/haic-eip/mugsi.html>. Medical record review is completed at each site by public health professionals trained specifically for clinical record abstraction. All abstractors undergo extensive EIP site-lead and CDC-lead training specific to the MuGSI CRF. To ensure accuracy of reported data, data quality checks were completed by CDC on all submitted data to validate more complex variables (e.g., culture source, associated infection types).

*Associated Infection Types*

The associated infection types reported by data collectors are based only on the information documented in the medical record. Urinary tract infection, including pyelonephritis, was selected on the CRF if it was clinically documented in the medical record in association with the uEc. Bacteremia was selected on the CRF if it was culture-proven in association with the uEc (e.g., *E. coli* positive blood culture collected the same date as the urine) or was clinically documented in the medical record in association with the uEc. Septic shock was selected on the CRF based on clinical documentation only (e.g., the phrase *septic shock* is included in a hospitalization history and physical or discharge summary).

*Epidemiologic Classification*

Epidemiologic classification defined as Hospital-onset: Urine obtained ≥ 3 days after hospital admission; Healthcare-associated, community-onset (HACO): Urine obtained in a non-hospital setting or < 3 days after hospital admission with selected healthcare exposures (hospitalization, surgery, residence in a long-term care facility or long-term acute care hospital) in the prior year, chronic dialysis, or with an indwelling device in the 2 days prior to culture; Community-associated: Urine obtained from a case-patient without any of the healthcare risk factors described.

*Whole Genome Sequencing*

Whole genome sequencing was conducted on all isolates received at CDC. Sequencing was performed using an Illumina MiSeq or NovaSeq system (San Diego, CA). Genomic DNA was extracted using the Promega Maxwell 48 Low Elution Volume DNA Purification Kit and the Maxwell 48 MDx Instrument (Madison, WI, United States). Libraries were prepared from genomic DNA using Illumina DNA prep reagents (Illumina, San Diego, CA) and barcoding indices synthesized in the CDC Biotechnology Core Facility. The libraries underwent QC, normalization and pooling, and the final pool was sequenced using Illumina Novaseq 6000 SP Reagent kit v1.5 (500 cycles) (Illumina, San Diego, CA). Sequencing reads were filtered for read quality, basecalled and demultiplexed using bcl2fastq (v2.20).

All sequences were analyzed using CDC’s PHoeNIx (v2.1.1) pipeline (Portable Healthcare Nextgen Informatics;  <https://github.com/CDCgov/phoenix>) to generate trimmed sequences and assemblies used for down-stream analyses.^1^

*In silico Serotyping*

*In silico* O serotyping was conducted using two serotyping tools, ECTyper v1.0.0^2^ and SerotypeFinder v2.0.2.^3^ Each tool was run using both the assemblies and trimmed sequences for each isolate. The O-serotypes were identified as the highest scoring type from both tools. O25 and O1 subtypes were identified by comparing the assembly against an in-house constructed subtyping database using GAMMA v2.1.^4^

| **eTable 1. Antimicrobial resistance of urinary *Escherichia coli* cases by patient sex and patient age based on reported antimicrobial susceptibility testing by local clinical laboratories, 3 U.S. counties, August 2023** | | | | | |
| --- | --- | --- | --- | --- | --- |
|  | **No. of resistant isolates/total No. isolates reported (%)** | | | | |
| **Resistance^a,b^ to Antimicrobial Agent** | **Overall** | **Female** | **Male** | **Age <60 Years** | **Age ≥ 60 Years** |
| **Aminoglycosides** | | | | | |
| Amikacin | 10/160 (6.3) | 7/130 (5.4) | 3/30 (10.0) | 2/76 (2.6) | 8/84 (9.5) |
| Gentamicin | 84/977 (8.6) | 77/828 (9.3) | 7/149 (4.7) | 45/528 (8.5) | 39/449 (8.7) |
| Tobramycin | 71/484 (14.7) | 64/394 (16.2) | 7/90 (7.8) | 32/259 (12.4) | 39/225 (17.3) |
| **Carbapenems** | | | | | |
| Ertapenem | 0/670 (0) | 0/556 (0) | 0/114 (0) | 0/331 (0) | 0/339 (0) |
| Imipenem | 0/114 (0) | 0/95 (0) | 0/19 (0) | 0/65 (0) | 0/49 (0) |
| Meropenem | 0/907 (0) | 0/768 (0) | 0/139 (0) | 0/498 (0) | 0/409 (0) |
| **Cephalosporins** | | | | | |
| Cefazolin | 160/945 (16.9) | 114/803 (14.2) | 46/142 (32.4) | 79/517 (15.3) | 81/428 (18.9) |
| Cefazolin (U)^c^ | 71/945 (7.5) | 47/803 (5.9) | 24/142 (16.9) | 29/517 (5.6) | 42/428 (9.8) |
| Cefepime | 9/332 (2.7) | 5/266 (1.9) | 4/66 (6.1) | 2/203 (1.0) | 7/129 (5.4) |
| Cefotaxime | 1/27 (3.7) | 0/21 (0) | 1/6 (16.7) | 1/16 (6.3) | 0/11 (0) |
| Cefoxitin | 2/78 (2.6) | 2/64 (3.1) | 0/14 (0) | 0/44 (0) | 2/34 (5.9) |
| Ceftazidime | 28/571 (4.9) | 16/470 (3.4) | 12/101 (11.9) | 12/290 (4.1) | 16/281 (5.7) |
| Ceftriaxone | 95/731 (13.0) | 61/608 (10.0) | 34/123 (27.6) | 44/385 (11.4) | 51/346 (14.7) |
| **Fluoroquinolones** | | | | | |
| Ciprofloxacin | 131/675 (19.4) | 100/576 (17.4) | 31/99 (31.3) | 56/335 (16.7) | 75/340 (22.1) |
| Levofloxacin | 132/631 (20.9) | 98/539 (18.2) | 34/92 (37.0) | 60/346 (17.3) | 72/285 (25.3) |
| **Folate pathway antagonists** | | | | | |
| Trimethoprim-sulfamethoxazole | 424/672 (63.1) | 346/558 (62.0) | 78/114 (68.4) | 220/363 (60.6) | 204/309 (66.0) |
| **Monobactams** | | | | | |
| Aztreonam | 1/13 (7.7) | 1/12 (8.3) | 0/1 (0) | 1/8 (12.5) | 0/5 (0) |
| **Nitrofuran** | | | | | |
| Nitrofurantoin | 13/839 (1.5) | 10/708 (1.4) | 3/131 (2.3) | 4/472 (0.8) | 9/367 (2.5) |
| **Penicillins** | | | | | |
| Ampicillin | 259/671 (38.6) | 215/570 (37.7) | 44/101 (43.6) | 136/340 (40.0) | 123/331 (37.2) |
| **Penicillins + β-lactamase inhibitors** | | | | | |
| Amoxicillin-clavulanate | 6/238 (2.5) | 6/208 (2.9) | 0/30 (0) | 2/130 (1.5) | 4/108 (3.7) |
| Ampicillin-sulbactam | 82/781 (10.5) | 66/660 (10.0) | 16/121 (13.2) | 36/407 (8.8) | 46/374 (12.3) |
| Piperacillin- tazobactam | 12/623 (1.9) | 9/522 (1.7) | 3/101 (3) | 5/327 (1.5) | 7/296 (2.4) |
| **Phosphonic acids** | | | | | |
| Fosfomycin | 9/13 (69.2) | 8/10 (80.0) | 1/3 (33.3) | 6/8 (75.0) | 3/5 (60) |
| **Tetracyclines** | | | | | |
| Tetracycline | 43/178 (24.2) | 31/143 (21.7) | 12/35 (34.3) | 23/92 (25.0) | 20/86 (23.3) |
| Minocycline | 0/22 (0) | 0/18 (0) | 0/4 (0) | 0/9 (0) | 0/13 (0) |
| ^a^Resistance determined by applying breakpoints for Enterobacterales based on the 2024 Clinical and Laboratory Standards Institute (CLSI) breakpoints (M100-ED34).^5^ | | | | | |
| ^b^Plazomycin, doripenem, meropenem-vaborbactam, imipenem-cilistatin-relebactam, cediferocol, ceftolozane-tazobactam, ceftazidime-avibactam, tigecyline, colistin, and doxycycline had no susceptibilities reported | | | | | |
| ^c^Cefazolin (U) refers to breakpoints for organisms isolated from the urinary tract | | | | | |

**eReferences**

1.	Hagey JV, Vlachos N, Kent AG, Masters T, Qi P, Halpin AL. CDCgov/phoenix: v2.1.1 (v2.1.1). Zenodo. https://doi.org/10.5281/zenodo.8147510

2.	Bessonov K, Laing C, Robertson J, et al. ECTyper: in silico Escherichia coli serotype and species prediction from raw and assembled whole-genome sequence data. *Microb Genom*. Dec 2021;7(12)doi:10.1099/mgen.0.000728

3.	Joensen KG, Tetzschner AM, Iguchi A, Aarestrup FM, Scheutz F. Rapid and Easy In Silico Serotyping of Escherichia coli Isolates by Use of Whole-Genome Sequencing Data. *J Clin Microbiol*. Aug 2015;53(8):2410-26. doi:10.1128/JCM.00008-15

4.	Stanton RA VN, de Man TJB, Lawsin A, Halpin AL. Development and application of QuAISAR-H: A bioinformatics pipeline for short read sequences of health care-associated pathogens. In: ASM Conference on Rapid Applied Microbial Next Generation Sequencing and Bioinformatic Pipelines. Tyson Falls, 2022.

5.	CLSI. *Performance Standards for Antimicrobial Susceptibility Testing*. 34th ed. CLSI supplement M100. Clinical and Laboratory Standards Institute; 2024.
